# Economic Burden of Cardiovascular Diseases in Brazil: Are Telemedicine and Structured Telephone Support the Solution?

**DOI:** 10.5935/abc.20180136

**Published:** 2018-07

**Authors:** Suzana Alves da Silva, Pedro Paulo Magalhães Chrispim, Yang Ting Ju, Ary Ribeiro

**Affiliations:** 1 Departamento de Epidemiologia do Hospital do Coração/Hcor, São Paulo, SP - Brazil; 2 Laboratório de Implementação do Conhecimento em Saúde do Hospital do Coração/Hcor, São Paulo, SP - Brazil; 3 Superintendência Comercial e de Serviços Ambulatoriais do Hospital do Coração/Hcor, São Paulo, SP – Brazil

**Keywords:** Cardiovascular Diseases, Health Policy, Cost-Effectiveness-Evaluation, Quality Management, Telemedicine/trends, Telephone/trends

The study by Stevens et al.^1^ results from a project of Delloite Consulting, financed by Novartis and aimed at estimating the economic burden that heart failure, acute myocardial infarction, atrial fibrillation and systemic arterial hypertension (SAH) impose on Latin American countries, and at assessing the cost-effectiveness of telemedicine and structured telephone support as interventions that can relieve it.^1^ The publication in this issue of the *Arquivos Brasileiros de Cardiologia* focused on presenting the results of the assessment in the Brazilian scenario.

This study provided us with the opportunity to reflect on important questions related to quality, interpretation and applicability of economic studies. Such studies have gained increasing relevance in the incorporation/disincorporation of technologies and the development of health policies and programs to improve healthcare quality. In addition, they are often used in other countries to support decision-making processes, although that is not a routine in Brazil.^2^

Several guidelines have been proposed in recent decades to improve the quality of the studies on economic assessment and their usefulness to healthcare systems. The *Consolidated Health Economic Evaluation Reporting Standards* (CHEERS)^3^ is a collection of those recommendations, recently updated and published in JAMA,^2^ which were only partially followed by Steven et al.

The measures used, for example, derived from sources not clearly indicated by the authors, who seem to have ignored any other related comorbidity besides the four conditions in question, such as stroke and chronic renal failure, as well as the presence or absence of other relevant comorbidities, such as diabetes, indicated by the NHS as one of the ten major causes of permanent disability and of high consumption of health resources currently.^4^ In addition, the differences in the levels of severity and heterogeneity between the Brazilian geographic regions seem not to have been considered. The incidence of sequelae and the rate of progression of those conditions resulting in morbidity, deaths and quality of life loss vary according to the intensity of the treatment provided, differing, thus, from region to region.^5-7^

The results reported by the studies in Venezuela^8^ and Mexico^9^ were neither cited nor discussed by the authors, although the cost-utility measures obtained were identical or very close in the three countries, suggesting that, at least partially, the data used were common to the three assessments.

The cost of primary attention seems to have been inferred from hospital expenditure data, assuming that the costs were equal. However, in at least one systematic review about the economic burden of heart failure, hospital expenditure was at least three times greater than outpatient clinic expenses, including the costs with procedures, tests and medicines.^10^

In addition, the prevalence estimates seem little accurate. According to Picon et al.,^11^ the prevalence of SAH has been decreasing by 3.7% every decade in Brazil. In the 1990s, the prevalence of SAH was estimated at 32.9%, while from 2000 to 2010, it was estimated at 28.7%, which would result in an expected prevalence from 2010 to 2020 lower than that observed in the previous decades. The authors started from a prevalence of 31.2% without indicating exactly what was the source of that information.

In the cost-effectiveness analysis, the interventions were not clearly defined, with disagreement between what the study claimed to assess (“telemedicine”) and the technology studied by the NHS report, on which the authors claimed to be based (“telemonitoring”).^12^ Especially for cost-effectiveness studies, depending on the intervention assessed, the results can be diametrically opposed, completely changing the recommendations.

In addition, according to the authors, the healthcare system costs attributable to those four conditions added up to 35 billion *reais* in 2015, which would represent one third of the total budget approved for health by the Brazilian Congress in that same year,^13^ suggesting that the estimates presented are overestimated.

Therefore, despite the relevance of the topic, the study by Stevens et al. provides convincing information on neither the burden of the selected diseases nor the cost-effectiveness of telemedicine or structured telephone support for approaching those conditions. The study has important limitations that prevents a clear interpretation of its results, as well as its application in the national scenario in a comprehensive manner.
